# Supplementary material for: Pyruvate Dehydrogenase Kinase 1 inhibition mediated oxidative phosphorylation enhancement in cartilage promotes osteoarthritis progression
Source: BMC Musculoskelet Disord. 2023 Jul 20;24:597. doi: 10.1186/s12891-023-06585-6 (PMC10357736; doi:10.1186/s12891-023-06585-6)
Supplement: Supplementary file 1 — Additional file 1. [file 12891_2023_6585_MOESM1_ESM.zip › BP.Gsea.1653623667859/GOBP_B_CELL_RECEPTOR_SIGNALING_PATHWAY.html]

Details for gene set GOBP\_B\_CELL\_RECEPTOR\_SIGNALING\_PATHWAY[GSEA]

|  || Dataset | 55457\_collapsed\_to\_symbols.cls.cls #OA\_versus\_normal.cls.cls #OA\_versus\_normal\_repos |
| Phenotype | cls.cls#OA\_versus\_normal\_repos |
| Upregulated in class | OA |
| GeneSet | GOBP\_B\_CELL\_RECEPTOR\_SIGNALING\_PATHWAY |
| Enrichment Score (ES) | 0.45949316 |
| Normalized Enrichment Score (NES) | 1.9995563 |
| Nominal p-value | 0.0 |
| FDR q-value | 0.021136517 |
| FWER p-Value | 0.378 |
Table: GSEA Results Summary

  

Fig 1: Enrichment plot: GOBP\_B\_CELL\_RECEPTOR\_SIGNALING\_PATHWAY      
 Profile of the Running ES Score & Positions of GeneSet Members on the Rank Ordered List

  

| SYMBOL | TITLE | RANK IN GENE LIST | RANK METRIC SCORE | RUNNING ES | CORE ENRICHMENT || 1 | BLNK | B cell linker [Source:HGNC Symbol;Acc:HGNC:14211] | 546 | 0.643 | -0.0102 | Yes |
| 2 | PTPN22 | protein tyrosine phosphatase non-receptor type 22 [Source:HGNC Symbol;Acc:HGNC:9652] | 578 | 0.630 | 0.0191 | Yes |
| 3 | IGHG1 | immunoglobulin heavy constant gamma 1 (G1m marker) [Source:HGNC Symbol;Acc:HGNC:5525] | 607 | 0.621 | 0.0482 | Yes |
| 4 | PLCG2 | phospholipase C gamma 2 [Source:HGNC Symbol;Acc:HGNC:9066] | 725 | 0.588 | 0.0687 | Yes |
| 5 | CD300A | CD300a molecule [Source:HGNC Symbol;Acc:HGNC:19319] | 730 | 0.587 | 0.0979 | Yes |
| 6 | BANK1 | B cell scaffold protein with ankyrin repeats 1 [Source:HGNC Symbol;Acc:HGNC:18233] | 802 | 0.568 | 0.1210 | Yes |
| 7 | BTK | Bruton tyrosine kinase [Source:HGNC Symbol;Acc:HGNC:1133] | 889 | 0.545 | 0.1418 | Yes |
| 8 | IGHV3-72 | immunoglobulin heavy variable 3-72 [Source:HGNC Symbol;Acc:HGNC:5622] | 1062 | 0.504 | 0.1538 | Yes |
| 9 | TRDC | T cell receptor delta constant [Source:HGNC Symbol;Acc:HGNC:12253] | 1100 | 0.496 | 0.1759 | Yes |
| 10 | PLCL2 | phospholipase C like 2 [Source:HGNC Symbol;Acc:HGNC:9064] | 1136 | 0.489 | 0.1978 | Yes |
| 11 | IGLC2 | immunoglobulin lambda constant 2 [Source:HGNC Symbol;Acc:HGNC:5856] | 1210 | 0.474 | 0.2160 | Yes |
| 12 | IGHA1 | immunoglobulin heavy constant alpha 1 [Source:HGNC Symbol;Acc:HGNC:5478] | 1426 | 0.439 | 0.2213 | Yes |
| 13 | TRBC1 | T cell receptor beta constant 1 [Source:HGNC Symbol;Acc:HGNC:12156] | 1458 | 0.435 | 0.2408 | Yes |
| 14 | IGKC | immunoglobulin kappa constant [Source:HGNC Symbol;Acc:HGNC:5716] | 1477 | 0.432 | 0.2612 | Yes |
| 15 | LAT2 | linker for activation of T cells family member 2 [Source:HGNC Symbol;Acc:HGNC:12749] | 1501 | 0.429 | 0.2810 | Yes |
| 16 | IGHV3-33 | immunoglobulin heavy variable 3-33 [Source:HGNC Symbol;Acc:HGNC:5596] | 1511 | 0.427 | 0.3018 | Yes |
| 17 | MS4A1 | membrane spanning 4-domains A1 [Source:HGNC Symbol;Acc:HGNC:7315] | 1652 | 0.406 | 0.3114 | Yes |
| 18 | CD79B | CD79b molecule [Source:HGNC Symbol;Acc:HGNC:1699] | 1729 | 0.394 | 0.3253 | Yes |
| 19 | FCGR2B | Fc fragment of IgG receptor IIb [Source:HGNC Symbol;Acc:HGNC:3618] | 2016 | 0.360 | 0.3211 | Yes |
| 20 | IGHM | immunoglobulin heavy constant mu [Source:HGNC Symbol;Acc:HGNC:5541] | 2241 | 0.335 | 0.3205 | Yes |
| 21 | LCK | "LCK proto-oncogene, Src family tyrosine kinase [Source:HGNC Symbol;Acc:HGNC:6524]" | 2274 | 0.329 | 0.3346 | Yes |
| 22 | CD79A | CD79a molecule [Source:HGNC Symbol;Acc:HGNC:1698] | 2288 | 0.328 | 0.3501 | Yes |
| 23 | IGHV3-21 | immunoglobulin heavy variable 3-21 [Source:HGNC Symbol;Acc:HGNC:5586] | 2404 | 0.314 | 0.3570 | Yes |
| 24 | CD38 | CD38 molecule [Source:HGNC Symbol;Acc:HGNC:1667] | 2416 | 0.312 | 0.3719 | Yes |
| 25 | IGHV1-69 | immunoglobulin heavy variable 1-69 [Source:HGNC Symbol;Acc:HGNC:5558] | 2461 | 0.307 | 0.3839 | Yes |
| 26 | NCKAP1L | NCK associated protein 1 like [Source:HGNC Symbol;Acc:HGNC:4862] | 2560 | 0.296 | 0.3911 | Yes |
| 27 | RFTN1 | "raftlin, lipid raft linker 1 [Source:HGNC Symbol;Acc:HGNC:30278]" | 2616 | 0.289 | 0.4014 | Yes |
| 28 | BLK | "BLK proto-oncogene, Src family tyrosine kinase [Source:HGNC Symbol;Acc:HGNC:1057]" | 2692 | 0.280 | 0.4097 | Yes |
| 29 | IGHV4-61 | immunoglobulin heavy variable 4-61 [Source:HGNC Symbol;Acc:HGNC:5655] | 2722 | 0.278 | 0.4214 | Yes |
| 30 | CD19 | CD19 molecule [Source:HGNC Symbol;Acc:HGNC:1633] | 2757 | 0.274 | 0.4326 | Yes |
| 31 | IGHV3-7 | immunoglobulin heavy variable 3-7 [Source:HGNC Symbol;Acc:HGNC:5620] | 2798 | 0.270 | 0.4431 | Yes |
| 32 | IGHV3-73 | immunoglobulin heavy variable 3-73 [Source:HGNC Symbol;Acc:HGNC:5623] | 3082 | 0.241 | 0.4332 | Yes |
| 33 | STAP1 | signal transducing adaptor family member 1 [Source:HGNC Symbol;Acc:HGNC:24133] | 3213 | 0.229 | 0.4346 | Yes |
| 34 | LPXN | leupaxin [Source:HGNC Symbol;Acc:HGNC:14061] | 3232 | 0.227 | 0.4446 | Yes |
| 35 | TRBC2 | T cell receptor beta constant 2 [Source:HGNC Symbol;Acc:HGNC:12157] | 3401 | 0.211 | 0.4422 | Yes |
| 36 | PTPN6 | protein tyrosine phosphatase non-receptor type 6 [Source:HGNC Symbol;Acc:HGNC:9658] | 3434 | 0.208 | 0.4502 | Yes |
| 37 | IGHV3-23 | immunoglobulin heavy variable 3-23 [Source:HGNC Symbol;Acc:HGNC:5588] | 3449 | 0.207 | 0.4595 | Yes |
| 38 | VAV3 | vav guanine nucleotide exchange factor 3 [Source:HGNC Symbol;Acc:HGNC:12659] | 4082 | 0.148 | 0.4177 | No |
| 39 | SYK | spleen associated tyrosine kinase [Source:HGNC Symbol;Acc:HGNC:11491] | 4084 | 0.147 | 0.4250 | No |
| 40 | MEF2C | myocyte enhancer factor 2C [Source:HGNC Symbol;Acc:HGNC:6996] | 4218 | 0.135 | 0.4215 | No |
| 41 | CD22 | CD22 molecule [Source:HGNC Symbol;Acc:HGNC:1643] | 4296 | 0.128 | 0.4220 | No |
| 42 | IGHV4-34 | immunoglobulin heavy variable 4-34 [Source:HGNC Symbol;Acc:HGNC:5650] | 4746 | 0.091 | 0.3916 | No |
| 43 | BCL2 | BCL2 apoptosis regulator [Source:HGNC Symbol;Acc:HGNC:990] | 5609 | 0.023 | 0.3256 | No |
| 44 | ITK | IL2 inducible T cell kinase [Source:HGNC Symbol;Acc:HGNC:6171] | 6008 | -0.005 | 0.2948 | No |
| 45 | MNDA | myeloid cell nuclear differentiation antigen [Source:HGNC Symbol;Acc:HGNC:7183] | 6100 | -0.013 | 0.2884 | No |
| 46 | BMX | BMX non-receptor tyrosine kinase [Source:HGNC Symbol;Acc:HGNC:1079] | 6265 | -0.025 | 0.2769 | No |
| 47 | IGHV5-51 | immunoglobulin heavy variable 5-51 [Source:HGNC Symbol;Acc:HGNC:5659] | 6399 | -0.035 | 0.2683 | No |
| 48 | PRKCB | protein kinase C beta [Source:HGNC Symbol;Acc:HGNC:9395] | 6453 | -0.039 | 0.2661 | No |
| 49 | LYN | "LYN proto-oncogene, Src family tyrosine kinase [Source:HGNC Symbol;Acc:HGNC:6735]" | 6514 | -0.043 | 0.2636 | No |
| 50 | MAPK1 | mitogen-activated protein kinase 1 [Source:HGNC Symbol;Acc:HGNC:6871] | 6853 | -0.068 | 0.2407 | No |
| 51 | IGHV3-20 | immunoglobulin heavy variable 3-20 [Source:HGNC Symbol;Acc:HGNC:5585] | 6859 | -0.068 | 0.2437 | No |
| 52 | PRKCH | protein kinase C eta [Source:HGNC Symbol;Acc:HGNC:9403] | 7141 | -0.092 | 0.2264 | No |
| 53 | PIK3CD | "phosphatidylinositol-4,5-bisphosphate 3-kinase catalytic subunit delta [Source:HGNC Symbol;Acc:HGNC:8977]" | 7405 | -0.111 | 0.2115 | No |
| 54 | IGLL1 | immunoglobulin lambda like polypeptide 1 [Source:HGNC Symbol;Acc:HGNC:5870] | 7544 | -0.122 | 0.2069 | No |
| 55 | PTPRC | protein tyrosine phosphatase receptor type C [Source:HGNC Symbol;Acc:HGNC:9666] | 7744 | -0.137 | 0.1983 | No |
| 56 | CD81 | CD81 molecule [Source:HGNC Symbol;Acc:HGNC:1701] | 8827 | -0.220 | 0.1251 | No |
| 57 | TEC | tec protein tyrosine kinase [Source:HGNC Symbol;Acc:HGNC:11719] | 8839 | -0.220 | 0.1353 | No |
| 58 | ABL1 | "ABL proto-oncogene 1, non-receptor tyrosine kinase [Source:HGNC Symbol;Acc:HGNC:76]" | 9081 | -0.238 | 0.1286 | No |
| 59 | BAX | "BCL2 associated X, apoptosis regulator [Source:HGNC Symbol;Acc:HGNC:959]" | 9323 | -0.257 | 0.1227 | No |
| 60 | GPS2 | G protein pathway suppressor 2 [Source:HGNC Symbol;Acc:HGNC:4550] | 9338 | -0.258 | 0.1347 | No |
| 61 | IGHD | immunoglobulin heavy constant delta [Source:HGNC Symbol;Acc:HGNC:5480] | 9396 | -0.263 | 0.1435 | No |
| 62 | IGHE | immunoglobulin heavy constant epsilon [Source:HGNC Symbol;Acc:HGNC:5522] | 10676 | -0.390 | 0.0635 | No |
| 63 | CTLA4 | cytotoxic T-lymphocyte associated protein 4 [Source:HGNC Symbol;Acc:HGNC:2505] | 10739 | -0.398 | 0.0787 | No |
| 64 | PLEKHA1 | pleckstrin homology domain containing A1 [Source:HGNC Symbol;Acc:HGNC:14335] | 10899 | -0.416 | 0.0873 | No |
| 65 | LIME1 | Lck interacting transmembrane adaptor 1 [Source:HGNC Symbol;Acc:HGNC:26016] | 11000 | -0.430 | 0.1011 | No |
| 66 | SH2B2 | SH2B adaptor protein 2 [Source:HGNC Symbol;Acc:HGNC:17381] | 12769 | -0.930 | 0.0102 | No |
Table: GSEA details [plain text format]

  

Fig 2: GOBP\_B\_CELL\_RECEPTOR\_SIGNALING\_PATHWAY      
 Blue-Pink O' Gram in the Space of the Analyzed GeneSet

  

Fig 3: GOBP\_B\_CELL\_RECEPTOR\_SIGNALING\_PATHWAY: Random ES distribution      
 Gene set null distribution of ES for **GOBP\_B\_CELL\_RECEPTOR\_SIGNALING\_PATHWAY**
